# Meta-analyses in cholestatic pregnancy: The outstanding clinical questions

**DOI:** 10.1177/1753495X241251425

**Published:** 2024-04-28

**Authors:** Nadejda Capatina, Caroline Ovadia

**Affiliations:** 1 Department of Women and Children’s Health, 4616 King's College London, UK; 2 East Suffolk and North Essex NHS Foundation Trust, UK

**Keywords:** Intrahepatic cholestasis of pregnancy, stillbirth, bile acid, meta-analysis, preterm birth

## Abstract

Reports of adverse pregnancy outcomes associated with maternal pruritus and liver impairment have circulated since the 1800s, yet the precise diagnosis and management of intrahepatic cholestasis of pregnancy have varied markedly. Recent evidence, including that from individual participant data meta-analyses, has provided an evidence that brings us closer to standardised, and optimal, management of the condition. Based upon increased adverse perinatal outcomes with higher bile acid concentrations, disease management should be according to severity (defined by peak bile acid concentration) in order to recommend appropriate gestation of birth. Similarly, the reduced spontaneous preterm birth rate for patients receiving ursodeoxycholic acid treatment suggests potential benefit for the treatment of patients with moderate-severe disease.

## Individual participant data meta-analyses in intrahepatic cholestasis of pregnancy

With a prevalence of between 0.5% and 5.6% (depending on population demographics and disease recognition),^1^ historic studies into intrahepatic cholestasis of pregnancy (ICP) have, by necessity, been relatively limited in size. A mechanism to address this can be to combine results from multiple previous studies, weighted in their contribution to the final effect size by the initial population size and outcome spread (confidence interval [CI]) in a standard meta-analysis, following a systematic review of the literature to identify all source data. Traditionally, systematic reviews and meta-analyses have been considered to provide the most reliable evidence (forming the ‘top layer’ of the pyramid of evidence quality).^2^ There has been an explosion in the number of systematic reviews and meta-analyses performed over recent years, as the availability of original manuscripts improved with online access and library subscriptions, the functionality of search engines, and systematic review-specific software improved.^3^ However, the quality of systematic reviews and meta-analyses is only as good as the source literature from which they take their data, and increasingly the risk of false conclusions being made from biased, or even falsified, work is being recognised.^4^ Retraction of such articles, when identified, may lead to alternative conclusions being reached.^5^

Reducing these risks is one benefit of individual participant data meta-analyses (IPD-MA). In an IPD-MA, authors are invited to contribute their individual results from each participant, rather than the summarised results typically presented in published results (e.g., mean, median, outcome rates). Further benefits of this approach are the ability to adjust for differing baseline variables consistently across the cohorts of patients included in all studies; to ensure that outcome definitions remain the same for all studies and to enable the inclusion of patient data collected but not reported in the source literature.

## Adverse perinatal outcomes

It was, therefore, an IPD-MA that has provided us with the most impactful data upon which evidence for adverse perinatal risks, and therefore management recommendations, has been based.^6^ This study (Ovadia et al., 2019) reported patient outcomes from 5269 pregnancies, from 25 published and 2 unpublished studies. Data were recovered from 15 countries in five continents, and represented approximately 50% of all reported studies identified through systematic review of the literature. The ‘headline’ finding was the clear demonstration of a bile acid threshold (100 µmol/L – defined as ‘severe’ disease) above which there was an increased risk of stillbirth in singleton pregnancies (prevalence 3.44%, 95% CI 2.05 to 5.37). This was more than 10-fold higher than for patients with peak bile acid concentration below 100 µmol/L, and significantly above background. In contrast, overall stillbirth rates were no higher than population rates for patients whose bile acids never increased above 100 µmol/L.

**Figure 1.**
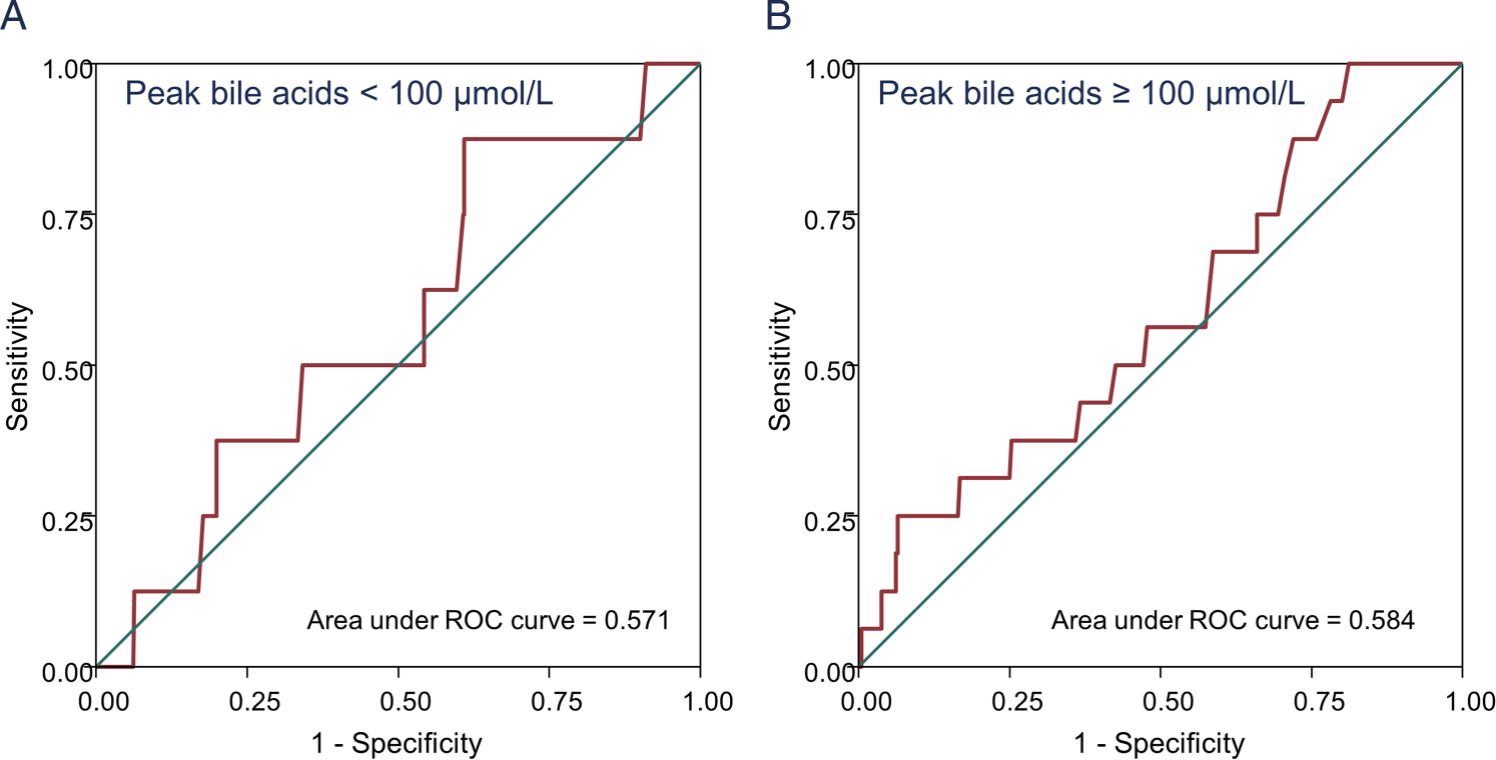
ROC curves demonstrating poor association between stillbirth and peak bile acid concentration in patients with peak bile acid concentration below (A) or above (B) 100 µmol/L. Data taken from contributors to the 2019 individual participant data meta-analysis detailing outcomes from pregnancies complicated by ICP.^6^

An alternative statistical approach to understand this threshold effect utilised a receiver operating characteristic (ROC) curve to understand the association between peak bile acid concentration and stillbirth. For the whole data set, the ROC area under the curve (AUC) for this was 0.85 (95% CI 0.77 to 0.93) – suggesting a good association between increased bile acid concentration and stillbirth risk. However, when ROC curves were separately produced for patients with peak bile acids below or above 100 µmol/L, there was no association between bile acid concentration and stillbirth risk (Figure 1). This suggests that, once bile acids are above 100 µmol/L, a further increase in bile acids is not associated with an increased risk of stillbirth. However, caution still should remain with interpretation, as these conclusions are based upon only 524 patients with bile acids above 100 µmol/L.

In addition to analysis of the risk of overall stillbirth, the IPD-MA enabled further interrogation of the risk of stillbirth by gestational week, according to peak bile acid category. Although the rate of stillbirth for each fetus in utero was marginally higher for patients with peak bile acids above 100 µmol/L than below from 24 weeks' gestation, it was from 35 weeks' gestation that the risk of stillbirth markedly increased for patients with severe disease, from 0.7% to 1.7%. This increase has stimulated UK and European recommendations to offer birth from 35 gestational weeks for patients with severe disease^7,8^; other national guidelines (Australia, New Zealand, US) have recommended offering birth from 36 weeks' gestation.^9,10^ It is difficult to envisage that the optimal week of birth to prevent stillbirth and minimise neonatal morbidity will ever be determined with clinical trial evidence, given the (fortunately) uncommon outcome of stillbirth and low proportion of patients with severe disease (approximately 10% of those with ICP have peak bile acids above 100 µmol/L).^6^ Evidence should be presented to patients as to the relative risks and benefits of birth at each gestational age and according to local healthcare (in particular, neonatal) services; decisions can therefore be achieved jointly between the clinician and the patient.

Previous work, led by Hanns-Ulrich Marschall, demonstrated that spontaneous preterm birth markedly increased when bile acid concentrations were over 40 µmol/L (moderate disease).^11^ The IPD-MA provided further evidence to support the management of patients with ICP.^6^ In particular, clear evidence demonstrated the markedly increased risk of preterm birth with ICP, both of spontaneous and iatrogenic (clinician-initiated) onset (odds ratio [OR] 3.47, 95% CI 3.06 to 3.95, and OR 3.65, 95% CI 1.94 to 6.85, respectively). These rates increased with higher bile acid elevation, with over 20% of babies being born prematurely when maternal bile acid concentrations peaked below 20 µmol/L, rising to nearly 60% when maternal bile acid concentrations were over 150 µmol/L. However, there was no specific threshold identified above which preterm birth significantly increased – potentially due to the influence of clinician-initiated preterm birth impacting the direct influence of serum bile acids on uterine contractility and preterm birth. At every bile acid concentration, iatrogenic preterm birth was more common than spontaneous. Similarly, within the IPD-MA cohort, there were increased rates of spontaneous preterm birth with moderate and severe disease (hazard ratios (HR) 1.34 (95% CI 1.06 to 1.69) and 2.77 (95% CI 2.13 to 3.61), respectively) compared with mild ICP (peak bile acid concentration below 40 µmol/L).^6^

There were no other biochemical markers reviewed in the IPD-MA that were highly predictive of stillbirth or preterm birth – with peak alanine aminotransferase (ALT), aspartate aminotransferase (AST) and bilirubin concentrations all investigated. Similarly, strong relationships (demonstrated by ROC AUC above 0.70) were not revealed between these markers or bile acid concentrations and other perinatal outcomes (meconium-stained amniotic fluid, non-reassuring fetal heart rate monitoring, Apgar score less than 7 at 5 min, umbilical cord arterial pH less than 7.0, neonatal unit admission or neonatal death).

## Impacts of treatment with ursodeoxycholic acid

The most commonly used disease-modifying treatment for ICP is ursodeoxycholic acid (UDCA); indeed, approximately two-thirds of the participants in the 2019 IPD-MA received UDCA.^6^ However, results of previous studies have been conflicting, with the most recent Cochrane meta-analysis reporting no clear evidence that UDCA improves perinatal outcomes or pruritus by a clinically meaningful degree.^12^ A further aggregate and IPD meta-analysis was performed (Ovadia et al., 2021), which included IPD from both randomised controlled trials (RCT) and observational studies.^13^ In contrast to the Cochrane review,^12^ comparator groups were combined for the RCT data (these included placebo, dexamethasone, S-Adenosylmethinoine, cholestyramine, and a combination of glucose, vitamin C and inosine, with or without phenobarbital), and a standard aggregate meta-analysis of these trials showed that UDCA treatment was associated with reduced overall preterm birth (OR 0.55, 95% CI 0.42 to 0.72), but not stillbirth (OR 0.44, 95% CI 0.08 to 2.31) or spontaneous preterm birth (OR 0.69, 95% CI 0.43 to 1.09). IPD were received from 4 of the 14 RCTs and 30 further observational studies, and comparisons adjusted for baseline bile acid concentration and parity. UDCA treatment was associated with significant reductions in a composite of stillbirth and preterm birth, all cause preterm birth, spontaneous preterm birth and meconium-staining of the amniotic fluid in singleton pregnancies. Fourteen patients would need to be treated with UDCA to prevent one preterm birth or stillbirth (95% CI 8 to 42). A major difference in the conclusions of the IPD-MA resulted from the additional data availability on spontaneous preterm birth risk not reported in all original manuscripts, demonstrating the additional benefit of such approaches.

In view of the research detailed above, the impact of UDCA on spontaneous preterm birth according to disease severity was assessed. Given the variabilities in management in observational studies, this analysis was limited to participants of the four RCTs providing IPD. In these studies, UDCA was clearly beneficial to reduce the risk of spontaneous preterm birth in patients whose bile acid concentration at the start of treatment was at least 40 µmol/L (HR 0.40, 95% CI 0.17 to 0.91), with a particular benefit for those with starting bile acid concentration between 40 and 100 µmol/L (HR 0.35, 95% CI 0.12 to 0.99).

In light of these findings, most international guidelines have recommended offering UDCA for patients with ICP to reduce the risk of adverse perinatal outcomes, despite the drug having limited effect on pruritus severity or total bile acid concentration.^8–10^ This is particularly relevant for patients with moderate disease (to reduce spontaneous preterm birth) or severe disease (to possibly reduce stillbirth). Given that UDCA has been consistently found to reduce liver aminotransferase concentrations,^12^ it may be of particular benefit for patients with markedly increased ALT or AST; although these are not directly associated with adverse outcomes, the difficulty to delineate ICP from other liver disorders presenting in pregnancy renders their reduction of likely benefit.

## What does this mean for the future?

The identification of clinically relevant thresholds for risks of adverse outcomes and treatment benefit will enable stratification of care according to peak bile acid concentration. Despite these recent advances, however, there remain many outstanding questions. Importantly, the significant findings from these IPD-MA were typically limited to patients with singleton pregnancies. Limited presented data from those with multifetal pregnancies increased the uncertainty in result interpretation (with wide confidence intervals or lack of sufficient outcome data for meaningful analysis).^6,13^ As such, we are currently completing a further IPD-MA in multifetal pregnancies and ICP.^14^ A further challenge to manage patients by serum bile acid concentration is that assays differ between sites, with studies reporting outcomes based on both enzymatic measurement and mass spectrometry within both IPD-MAs previously discussed.^6,13^ Similarly, assay performance can vary between laboratories; harmonisation of assays provides a potential solution utilising mathematical modelling as an alternative to QC utilising measurement of standards of known concentration across sites.^15^

Similarly, are there better ways to understand which babies from pregnancies with severely increased bile acids are at risk of stillbirth? That a biological threshold exists above which stillbirth risk is increased suggests a mechanism of stillbirth associated with sudden compromise in fetal function not compatible with life. Given that chronic hypoxia and intrauterine growth restriction are not an established feature of ICP,^6^ suggested mechanisms of ICP-related stillbirth have included fatal fetal arrhythmia or sudden placental vessel vasospasm.^16,17^ Evidence for the former arises from both *in vitro* and clinical studies. Cardiomyocytes can be cultured, and will develop synchronous electrical activity from a stable originating point; adding taurocholic acid, the bile acid most increased in ICP, triggers disorderly electrical currents and even patterns evocative of a tachyarrhythmia.^16^ Interestingly, this appears to be reversed by addition of UDCA.^18^ This experimental hypothesis is supported by recordings from fetal electrocardiograms performed during ICP pregnancies, which reveal prolonged PR intervals and altered heart rate variability in patients with severe ICP.^19^ Again, these changes were not seen in patients treated with UDCA. Similarly, UDCA may be of benefit to reduce placental vasospasm secondary to taurocholic acid, by inhibiting organic anion-transporting polypeptide (OATP)4A1-mediated placental bile acid transfer and taurocholic acid-induced placental vasoconstriction.^20^

As our management of patients with ICP is now stratified by peak bile acid concentration, can we predict which patients will develop moderate or severe disease from the point of diagnosis? It is well recognised that the pruritus associated with ICP can develop before hypercholanaemia occurs; the median duration between pruritus onset and first increase of bile acids was around 14 days.^21^ In this study, serum autotaxin activity and concentrations of two disulfated progesterone metabolites were predictive of subsequent ICP. However, these tests are not routinely available and did not address predictive potential for these markers for later disease severity.

Once ICP is diagnosed, then the benefit of UDCA to reduce adverse perinatal outcomes is becoming clear.^13^ However, alternative treatments are required to improve the associated pruritus, and reduce serum bile acid concentrations. A possible candidate for pharmacological management of ICP is the antibiotic rifampicin, which inhibits bile acid synthesis as an agonist of pregnane X receptor. Rifampicin use has been associated with reduced pruritus and bile acid concentrations in an observational cohort of its use in 27 women with ICP.^22^ In light of these findings, its use is being investigated in the TURRIFIC randomised trial in ICP, in comparison with UDCA.^23^

Recently, newer pharmaceutical agents have been developed targeting aspects of bile acid metabolic pathways. An intestinal bile acid transport (IBAT) inhibitor, volixibat, was studied in women with ICP – this blocks reuptake of bile acids in the intestine, thereby increasing faecal bile acid excretion. Although the study was terminated early, initial results have been reported and appear promising^24^; IBAT inhibitor use is progressing in other cholestatic conditions.^25^ Similarly, non-gestational cholestasis provides an alternative disease model for potentially useful drug agents to be investigated: EP547 is an antagonist for the MRGPRX4 receptor found on skin nerve cells thought responsible for bile acid-mediated itch, and is currently being trialled in the PACIFIC study of autoimmune cholestasis.^26^

## How can we support future research?

**Figure 2.**
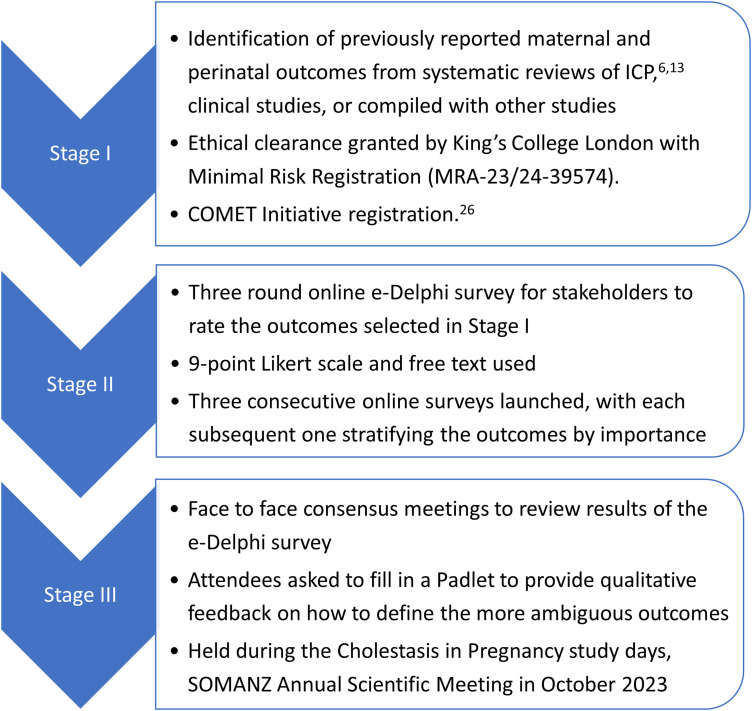
Protocol for Delphi consensus to define a core outcome set for reporting future studies of ICP.

Whilst collecting data for the IPD-MA, what became clear was the inconsistencies in outcomes reported and their definition, which limited between-study comparisons. To assist, we are therefore establishing a consensus to develop a Core Outcome Set to be included in future studies of ICP.^27^ This has used a Delphi process to include opinions from clinicians, scientists and patients, with a pre-specified protocol registered with the COMET initiative (Figure 2). Following a systematic review of the literature, reported clinical outcomes were collated and separated into maternal, neonatal and birth outcomes. Participants were requested to score the importance of each outcome according to a Likert score (0–9); these results were presented to the participants, who then rescored the outcome importance. Following selection of the most important outcomes in a third online round, two ‘in person’ consensus meetings were held as part of the SOMANZ 2023 Cholestasis in Pregnancy Expert and Consumer days. Delegates ranked the final 23 outcomes by importance, and provided qualitative feedback on definitions for the more ambiguous outcomes. Finally, the steering committee will select the appropriate outcomes for the core outcome set based upon this international consensus – completion of which is imminent.

## Conclusions

There is no doubt that IPD-MA are highly effective in maximising the research efficiency of individual studies, particularly when studying rare outcomes in uncommon diseases. Similarly, they encourage internationally collaborative work, and support researchers to contribute to highly impactful work to improve the care of affected patients. By including studies from a range of clinical settings and countries, results from IPD-MA may be more accepted as reflective of populations in multiple locations. Continuing international collaboration will remain important to ensure that future work can provide timely evidence as to the impact of recent advances in ICP. Similarly, we encourage researchers to collect standardised data to increase meaningful future comparisons, and eagerly anticipate completion and dissemination of the Core Outcome Set in ICP to support this work.
